# Exploring Risk Constellations for Noncommunicable Diseases: A Community-Based Study From Northern India

**DOI:** 10.7759/cureus.96043

**Published:** 2025-11-03

**Authors:** Vijna, Chandra Pati Mishra

**Affiliations:** 1 Community Medicine, Baba Kinaram Autonomous State Medical College, Chandauli, IND; 2 Community Medicine, Heritage Institute of Medical Sciences, Varanasi, IND

**Keywords:** and intermediate risk factors, behavioural risk factors, non-communicable diseases, non-modifiable risk factors, risk constellation

## Abstract

Background: Noncommunicable diseases (NCDs) have emerged as a leading public health challenge in rural India, largely driven by the clustering of non-modifiable, behavioral, and intermediate risk factors.

Methodology: To determine the distribution and clustering of NCD risk factors, a community-based cross-sectional study was conducted among rural adults from three villages, namely, Umraha, Narayanpur, and Chittauna, in the Chiraigaon block of Varanasi district, North India. Using a multistage, stratified random sampling technique, 425 individuals aged 25-64 years were selected. Data collection was carried out over one year, from February 2019 to January 2020, using a culturally adapted WHO STEPwise questionnaire.

Results: Behavioral risks were highly prevalent, including inadequate fruit and vegetable intake (*n* = 424, 99.8%), physical inactivity (*n* = 387, 91.1%), excess dietary salt consumption (*n *= 354, ~83%], tobacco use (*n *= 156, 36.7%), and alcohol intake (*n *= 93, 21.9%). The intermediate risks included hypertension (*n *= 134, 31.5%), central obesity (*n *= 123, 28.9%), and overweight/obesity (*n *= 108, 25.4%). A family history of NCDs was reported by 225 (52.9%) participants. Risk factor clustering revealed that no individual was risk-free; 228 (53%) individuals were classified as high-risk (≥5 risk factors), and the median number of risk factors was 5. Multivariate logistic regression identified age as the strongest predictor of high-risk status, with adults aged 45-64 years having nearly fourfold higher odds than those aged 25-44 years (adjusted odds ratio (AOR) 3.95; 95% confidence interval (CI): 2.36-6.61). Lower educational attainment also significantly increased the odds of being at high risk (*P *< 0.05), while gender and occupation lost significance in the adjusted models.

Conclusions: Substantial clustering of modifiable NCD risk factors in this rural setting emphasizes the urgent shift from a conventional high-risk approach to community-led macro-level blanket intervention.

## Introduction

In contemporary global landscapes, noncommunicable diseases (NCDs) stand out as the most challenging health concerns. Annually, 43 million deaths, that is, 75% of global deaths, are attributed to the NCD menace [1]. Low- and middle-income countries account for 86% and 75% of all premature NCD deaths and total NCD deaths, respectively [1-3]. This predicament is also reflected in the World Health Organization (WHO), National NCD Monitoring Survey (NNMS), and ICMR-INDIAB (Indian Council of Medical Research-India Diabetes Study) report on India’s NCD profile [4-8]. India is confronting an epidemiological shift, that is, infectious diseases are receding [9] and chronic diseases are surging, thereby facing a double burden [10]. NCDs are responsible for over 68% of all deaths in India [11].

There is a complex interplay of risk quartets, namely, non-modifiable factors (age, sex, gender, genetic predisposition, and family history), behavioral factors (tobacco use, unhealthy diets, harmful use of alcohol, and insufficient physical activity), metabolic/ intermediate factors (raised blood pressure, overweight/obesity, hyperglycemia, and hyperlipidemia), and environmental risk factors (air pollution) [1]. Hence, NCDs are considered to be multifactorial [12]. While behavioral risk factors are receptive to change [13], intermediate factors represent more permanent physiological damage, which often results from persistent exposure to behavioral risk factors [4]. Non-modifiable risk factors, on the other hand, are intrinsic and unaltered.

A wealth of literature has documented the prevalence of risk factors across diverse scenarios. For example, under the behavioral risk factor trait, tobacco alone kills more than 7 million people annually, including 23% of second-hand smokers [14]. The Global Adult Tobacco Survey (GATS) in India showed 10.7% to 21.4% of smoke and smokeless tobacco use, respectively [15]. The Fifth National Family Health Survey also reported use of tobacco as 39% and 4% in males and females, respectively [16]. Approximately 2.6 million deaths per year, i.e., one out of 21 deaths, are attributed to alcohol by the WHO [17]. In India, alcohol consumption ranges from 22.9% in males to 0.7% in females [16]. It is also estimated that, of all the disease burden in India, 56.4% is due to unhealthy diets [18]. Too much sodium consumption in the diet also causes an estimated 1.89 million deaths per year [19]. Altered lifestyles, changing occupational patterns, and increased screen time give rise to a pervasive issue of physical inactivity [20], which, in turn, increases disease risk in 1.8 billion individuals [21]. Globally, among metabolic risk factors, elevated blood pressure is the leading cause of death (25% of global NCD deaths are attributed to it) [22]. The prevalence of adult hypertension in India is 21% and 24% in women and men, respectively [16]. Worldwide, 1 in 7 individuals live with diabetes; among them, 77 million are Indian [23,6]. In 2022, 43% adults were overweight, including 12.5% people living with obesity [24,25], whereas 3.7 million people died due to higher body mass index [26]. Turning to non-modifiable risk factors, age above 45 years and male gender sharply increase mortality and disability-adjusted life years (DALYs). These risk factors configure the macro-level distribution of NCDs [27] and impose substantial strain on a country’s economic productivity and healthcare system [10].

While a multitude of studies have singularly explored NCDs and their associated risk factors, a concurrent charting of the "risk constellation framework" within a particular community is crucial for the development of strategically diverse health interventions. In contrast to their urban counterparts, the rural population displays an array of distinct profiles, namely, sociocultural barriers, occupational constraints, healthcare access ambiguity, and health information paucity [28]. This study aims to bridge this gap and to find the distribution and determinants of high-risk groups (a constellation of behavioral, intermediate, and non-modifiable risk factors) contributing to NCDs among rural adults. The novelty of this study lies in its holistic perceptive. Evidence from this study will provide insights into programmatic action points to curb the rising NCD epidemic.

## Materials and methods

This cross-sectional study was conducted in a rural community in North India to assess the distribution and determinants of the high-risk groups associated with NCDs. The study was conducted between January 2019 to October 2020, with data collection carried out over a period of one year, from February 2019 to January 2020. A multistage stratified random sampling approach was adopted, and 425 adult participants were enrolled. This sample size was obtained by taking the prevalence of the high-risk category as 46.7%, obtained from an unpublished pilot study conducted in a non-study area.

In the first stage, Chiraigaon Community Development Block was randomly selected from the eight blocks of the Varanasi district using the lottery method. Within the selected block, villages were stratified according to their distance from the block headquarters (<5 km, 5-10 km, and >10 km). One village from each stratum was then chosen randomly using the lottery method, based on the official village list obtained from block records. Accordingly, Umraha, Narayanpur, and Chittauna were selected from the first, second, and third strata, respectively. The number of households included from these villages was 144, 169, and 112. It was determined by using the probability proportional to size (PPS) sampling method, corresponding to their respective populations of 6,429, 7,517, and 5,011. Within each selected village, systematic random sampling was applied to identify households, and from each chosen household, one adult aged 25-64 years was selected by the lottery method. Adults who had been residing in the selected area for at least six months were included in the study. Individuals with severe illness, cognitive impairment, or unwilling to provide informed consent were excluded.

Tools and techniques

A semi-structured interview schedule served as the tool for this study. This tool is an adaptation of an original work *STEPwise Approach to Surveillance (STEPS) of Noncommunicable Disease Risk Factors*. Geneva: World Health Organization (WHO); 2017. License: CC BY-NC-SA 3.0 IGO [29]. This adaptation was not created by WHO. WHO is not responsible for the content or accuracy of this adaptation. The original edition shall be the binding and authentic edition. The tool was pre-tested and adapted to ensure contextual and cultural salience. It includes sections on the sociodemographic profile and three risk categories: behavioral (e.g., tobacco use, alcohol consumption, physical inactivity <150 minutes/week, unhealthy diet <5 servings of fruits and vegetables per day, and self-reported additional salt consumption, which may not reflect total dietary sodium intake but serves as a proxy for high-salt intake behavior); intermediate (e.g., hypertension, diabetes, overweight, obesity, and abdominal obesity); and nonmodifiable (e.g., age, sex, and family history of NCDs).

Calibrated instruments and standard protocols were used for anthropometric measurements (height, weight, waist, and hip circumferences). Cutoff values for various parameters were set according to standard guidelines. For instance, waist-to-hip ratio (WHR) cutoffs were ≥0.90 for males and ≥0.85 for females; Asian-specific BMI cutoffs for overweight and obesity were 23.0-24.9 kg/m² and ≥25 kg/m², respectively; and blood pressure cutoffs were systolic blood pressure (SBP) ≥140 mmHg or diastolic blood pressure (DBP) ≥90 mmHg. Current medication history and self-reported history of diabetes were used as risk factor indicators amidst the unavailability of field-based biochemical evaluation.

Operational definitions

Risk categories were calculated by summing each risk trait in an individual, and dichotomized into *low* (≤4 risk factors) and *high* (≥5 risk factors) risk categories.

Statistical analysis

Data were analyzed using IBM SPSS version 25 (IBM Corp., Armonk, NY). Descriptive statistics such as frequency, percentage, mean, and standard deviation (SD) were used to summarize the data. The association was established using chi-square tests. Multivariable logistic regression was used to identify the predictors of high-risk categories for NCDs.

## Results

A total of 425 participants were included in the study. The largest proportion of participants (*n *= 167, 39.3%) was between 25 and 34 years of age, and 124 (29.2%) subjects were in the 35-44 years age group. The remaining 92 (21.6%) and 42 (9.9%) individuals of the study sample were between the ages of 45 and 54 years and 55 to 64 years, respectively. The sample showed a slight male predominance (*n* = 225, 52.9%), with the majority of the participants being Hindu (361, 84.9%), and Muslims accounted for 64 (15.1%) individuals. The majority of participants belonged to Other Backward Classes (OBC; 224, 52.7%), followed by 104 (24.5%) from Scheduled Castes (SC), 96 (22.6%) from the General category, and only 1 (0.2%) from Scheduled Tribes (ST).

Nearly 351 (83%) of respondents were married, while smaller proportions were unmarried (37, 8.7%), widowed (26, 6.1%), separated (4, 0.9%), or deserted (7, 1.6%). Educational attainment ranged widely, with 38 (8.9%) illiterate, 63 (14.8%) literate, and 20 (4.7%) having pursued graduation or higher education. In terms of schooling, most individuals had completed primary school (90, 21.2%), middle school (83, 19.5%), high school (70, 16.5%), or intermediate school (50, 11.8%). Approximately one in three respondents were homemakers (148, 34.8%), 107 (25.2%) were laborers, 73 (17.2%) were agriculturists, 45 (10.6%) were engaged in business, and a small proportion (22, 5.2%) worked in formal service sectors. Additionally, 9 (2%) individuals were pursuing higher education, and 21 (5%) were unemployed.

**Figure 1:**
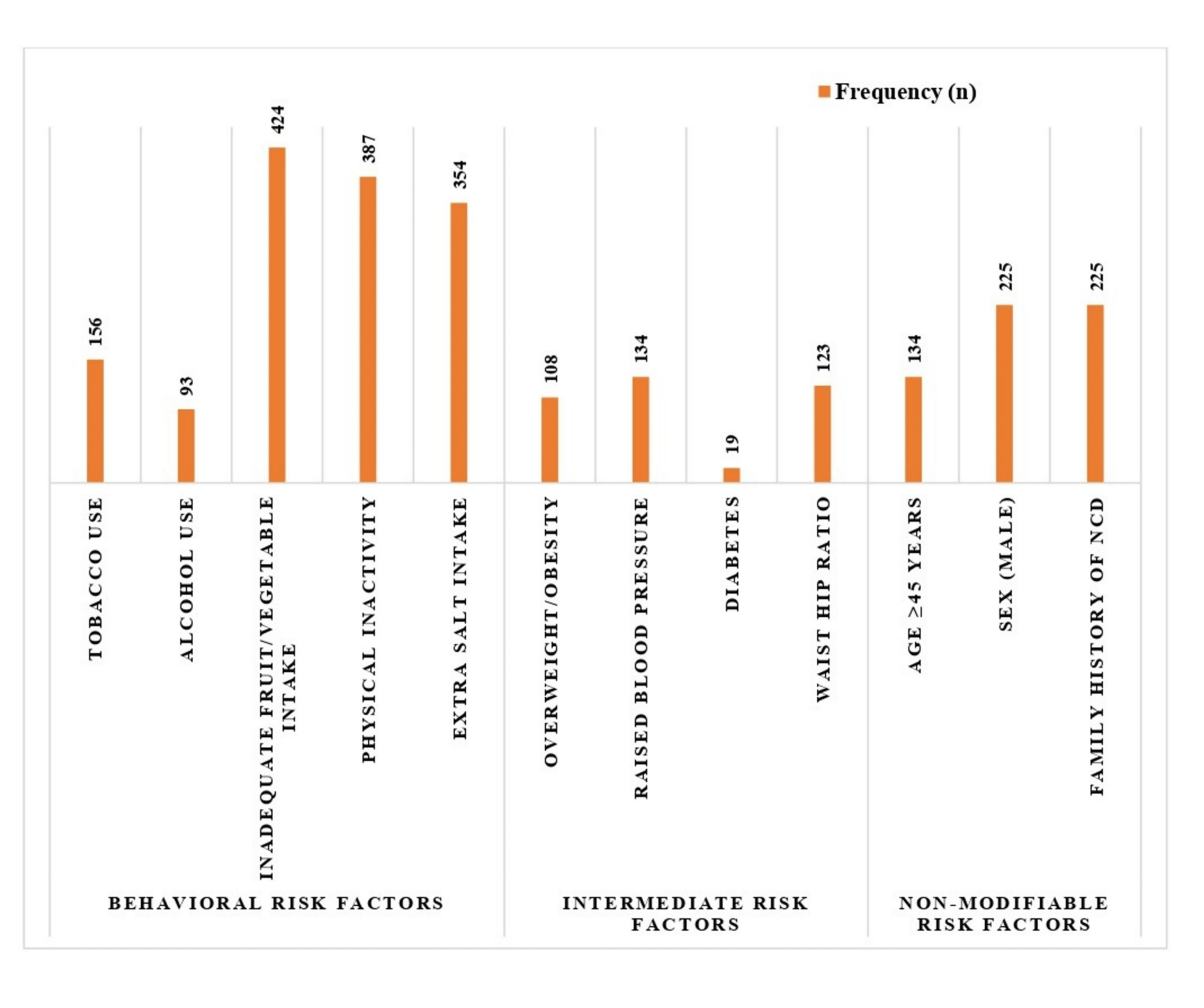
Distribution of risk factors among participants (N = 425).

The distribution of the risk factors among the study participants is shown in Figure 1. In the behavioral risk domain, a substantial number of participants (424, 99.8%) reported inadequate fruit and vegetable intake. Similarly, physical inactivity was observed in 387 (91.1%) respondents, and approximately five out of six individuals admitted to consuming extra dietary salt. The reported use of tobacco and alcohol in the study population was 156 (36.7%) [123 (54.7%) in males and 33 (16.5%) in females] and 93 (21.9%) [92 (40.9%) in males and 1 (0.5%) in females], respectively. There was also a noticeable prevalence of intermediate-risk factors. Approximately one in four individuals (108, 25.4%) were overweight or obese. Raised blood pressure was reported in 134 (31.5%) individuals, 123 (28.9%) had an elevated waist-to-hip ratio, and diabetes was observed in 19 (4.5%) of study participants. Under the non-modifiable risk profile, 134 (31.5%) participants were aged 45 years or above, males represented 225 (52.9%) of the total sample, and a similar prevalence, 225 (52.9%), was reported for a positive family history of NCDs.

Information about risk factor clustering is presented in Table 1. In the context of risk factor clustering, no participant was risk-free. Nearly 198(47%) of the individuals were in the low-risk category, and 228(53%) were in the high-risk category. Approximately 139(33%) of the participants reported the most frequent risk cluster of 4. The risk distribution extended from a minimum of 2 risk factors to a maximum of 8 risk factors per individual. Participants exhibited multiple NCD risk factors, with a median number of risk factors of 5 and a mean of 4.72 ± 1.20.

**Table 1:** Distribution of subjects according to number of risk factors.

Risk category	Number of risk factors present per individual	Frequency (*n*)	Percentage (%)
Low risk	1	0	0.0
	2	6	1.4
	3	53	12.5
	4	139	32.7
High risk	5	124	29.2
	6	68	16.0
	7	28	6.6
	8	7	1.6
	9	0	0.0

The associations between various personal attributes and risk group categories are given in Table 2. There was a significant association between the NCD risk status of subjects and their age, gender, and educational as well as occupational status. The prevalence of high-risk categories for NCDs was higher among participants aged 45-64 years (*n* = 101, 75.4%) and male participants (*n* = 132, 58.7%). A total of 73 (65.2%) and 127 (52.3%) individuals from the illiterate/literate/just literate and primary/middle/high school educational categories, respectively, had a high NCD risk status; the corresponding value for those with intermediate and higher education was 38.6% (*P* < 0.001). Participants with the lowest educational levels (illiterate/literate/just literate) were significantly more likely to have a high-risk profile (χ² = 12.56, *P* < 0.001) than those in other educational categories. Individuals engaged in income-generating occupations had a higher proportion in the high-risk category (*n* = 143, 57.9%), which was significantly different (χ² = 4.76, *P* = 0.03) from those not involved in such work (*n* = 84, 47.2%). Independent variables such as religion, caste category, marital status, and family history of NCDs had no significance in risk group categorization.

**Table 2:** Associates of noncommunicable disease (NCD) risk groups. SC/ST, Scheduled Caste/Scheduled Tribe; OBC, Other Backward Classes

Variables	Category		Total (*N*)	Low risk, *n* (%)	High risk, *n* (%)	χ²	df	*P*-value
Sociodemographic profile, including non-modifiable factors	Age group	<45 years	291	165 (56.7%)	126 (43.3%)	37.93	1	0.00
		≥45 years	134	33 (24.6%)	101 (75.4%)			
	Gender	Male	225	93 (41.3%)	132 (58.7%)	5.31	1	0.02
		Female	200	105 (52.5%)	95 (47.5%)			
	Religion	Hindu	361	167 (85.7%)	194 (86.0%)	0.10	1	0.75
		Muslim	64	31 (48.4%)	33 (51.6%)			
	Caste	SC/ST	107	47 (44.8%)	58 (55.2%)	0.36	2	0.84
		OBC	224	104 (46.4%)	120 (53.6%)			
		General	96	47 (49.0%)	49 (51.0%)			
	Marital status	Married	351	165 (47.0%)	186 (53.0%)	0.14	1	0.71
		Single/Widow/Other	74	33 (44.6%)	41 (55.4%)			
	Educational status	Intermediate and above	70	43 (61.4%)	27 (38.6%)	12.56	2	0.00
		Primary/Middle/High school	243	116 (47.7%)	127 (52.3%)			
		Illiterate/literate/just literate	112	39 (34.8%)	73 (65.2%)			
	Occupational status	Engaged in gainful income	247	104 (42.1%)	143 (57.9%)	4.76	1	0.03
		Not engaged in gainful income	178	94 (52.8%)	84 (47.2%)			
	History of NCD	Yes	225	107 (47.6%)	118 (52.4%)	0.18	1	0.67
		No	200	91 (45.5%)	109 (54.5%)			

Multivariate logistic regression analysis was performed to identify the independent predictors of high-risk groups. Age was found to be the strongest predictor. In comparison to the age group of 25-44 years, subjects belonging to the age group of 45-64 years had a higher AOR (3.95; 95% CI: 2.36-6.61) for the high-risk category, that is, participants aged 45-64 years had nearly four times the odds of being in the high-risk group compared to those aged 25-44 years. There was also a significant association between educational divisions and high-risk categories. Taking intermediate or higher education as reference, illiterate, literate, and literate (AOR = 2.13, 95% CI: 1.03-4.39; *P* = 0.04) had twice the odds of high-risk status. Similarly, individuals with education up to high school (AOR = 1.91, 95% CI: 1.06-3.43, *P* = 0.03) exhibited increased odds of being in the high-risk group than the reference intermediate and above. The significant association of sex and occupation of subjects with their risk status of NCDs obtained in bivariate analysis was eliminated in the multivariate logistic regression analysis (Table 3).

**Table 3:** Findings of logistic regression analysis regarding predictors of NCD risk status in subjects. *Reference category. NCD, noncommunicable disease; AOR, adjusted odds ratio; CI, confidence interval

Particulars		Estimation of β	SE of β	*P*-value	AOR	95% CI	
						Lower	Upper
Age (in years)	45-64	1.37	0.26	0.00	3.95	2.36	6.61
	25-44*	-	-	-	-	-	-
Gender	Male	0.32	0.31	0.30	1.38	0.75	2.54
	Female*	-	-	-	-	-	-
Education	Illiterate+ Just Literate+ Literate	0.76	0.37	0.04	2.13	1.03	4.39
	Primary+ Middle+ High school	0.65	0.29	0.03	1.91	1.06	3.43
	Intermediate and above*	-	-	-	-	-	-
Occupation	Engaged in gainful employment	0.51	0.31	0.10	1.67	0.91	3.07
	Not engaged in gainful employment*	-	-	-	-	-	-

## Discussion

This study elucidates the prevalence and determinants of risk categories in the adult population of northern India. Approximately seven out of 10 individuals are under 45 years of age, which mirrors India's youthful demography [30]. NCDs typically manifest later in life. However, their risk factors are increasingly prevalent in the younger population. This particular demography signals a lurking NCD epidemic in India, probably a decade before the developed nations [31,32]. The education profile comprises 26.3% illiterate, just literate, and literate individuals, and 0.2% with postgraduate degree and above holders, which is in alignment with lower educational profiles in many North Indian states [33]. These figures contrast with India’s 2017 national literacy rate of approximately 77.7% [34]. Comprehension and adoption of preventive healthy behaviors are hindered by limited formal education [35]. Consistent with the regional demographics of Northern India, the study population comprised 84.9% Hindus and 15.1% Muslims. The caste distribution showed a predominance of OBC (52.7%), followed by SC (24.5%), General (22.6%), and ST (0.2%), reflecting the patterns reported in the 2011 Socioeconomic and Caste Census [36,37]. Socioeconomic status, health information propagation, and healthcare access are strongly influenced by this dual construct of Indian identity (Religion and Caste). Occupational distribution also represents the rural and semi-urban socioeconomic landscape of Northern India [38].

Understanding the distribution of behavioral risk factors, alarmingly, inadequate fruit and vegetable intake was observed in 99.8% of participants. This exceeds significantly from the insights of different national estimates [39-41]. Economic constraints and limited nutritional knowledge could be the drivers of this systemic dietary shift from traditional balanced diets. This pattern escalates NCD vulnerability, reinforcing the urgent need for nutritional education.

A study in Tamil Nadu (40.6%) [42] and South India (46.8%) [43] showed markedly lower physical inactivity than our study (91.1%). It is paradoxical for agriculturists, laborers, and traditional homemakers to show high inactivity in this rural setting, probably driven by altered work patterns and mechanization. Hence, an active lifestyle should be promoted beyond occupational tasks. The high prevalence of extra dietary salt intake (83.3%) also aligns with the national trends. India’s daily salt intake (11-12 grams) is almost double that recommended by the WHO [44]. Awareness campaigns about the health risk of excessive salt consumption fail to impact the habit of serving salty food as an affordable flavoring agent in this region. In this study, three out of eight and two out of nine participants were tobacco and alcohol consumers, respectively. Similar trends across different substance use in rural settings have also been reflected in NFHS 5 [16].

Among intermediate risk factors, increased blood pressure affected 3 out of 10 individuals, which was found to be consistent with a review by Kario et al. [45]. This figure also exceeded the NFHS-5 reported prevalence (21% for women, 24% for men) [16]. Congruent with the findings of the NAMS Task Force(33), NFHS-5 (six out of 25 women and three out of 13 men nationally) [16], and a recent press release by the Government of India [46], our study also stated that nearly one in four subjects was overweight and obese. Central obesity was more than normal in nearly one out of three individuals. Other studies, such as the PERSIAN Guilan cohort study and Longitudinal Aging Survey on older adults (45+ years), also reported significantly higher WHR prevalence (24 out of 33 and 7 out of 9 individuals, respectively). This is a concerning difference pertaining to the younger age of our study cohort [47,48]. The emerging diabetes prevalence (1 out of 22) indicates a crisis. Granular analysis of the ICMR-INDIAB study for the Uttar Pradesh state also reported nearly one out of 20 individuals as diabetics [49].

Overall, no participant was NCD risk-free, and similar findings have also been reported from the findings of Puducherry district, highlighting the profound public health significance [50]. Revelations from our study are extreme compared to the findings from a South Indian district, which stated that around 7 out of 50 from the urban and nearly 9 out of 50 from the rural population had none of the seven NCD risk factors [51]. This highlights the strategic shift of NCD management from a conventional high-risk approach to a broader stroke roadmap. Furthermore, five or more NCD risk factors were observed in nearly 11 out of 20 participants, and the average risk factor count was 4.72 ± 1.20. Two other studies from South India, contrastingly, reported a lower fraction of clustering (i.e., nearly one out of five with ≥ 5 risk factors and 4 out of 15 with five risk factors, respectively) [43,50].

Regardless of the predominance of a young study population, age (AOR = 3.95 for 45-64 years) surfaced to be the strongest predictor of high-risk status for NCDs. This is in alignment with the worldwide epidemiological dynamics that the prevalence of NCDs generally increases with age [1]. This underlines that, while risk factors are rampant in younger groups, their compounded effect becomes more evident with progressing age. The lowest literacy spectrum (AOR = 2.13, *P* = 0.04) and education up to high school (AOR = 1.91, *P* = 0.03) further reflected higher odds for the high NCD risk category, which underscores the protective effect of education. Paucity in formal education is, in turn, linked to reduced pro-health behaviors [52]. Contrarily, data from the Longitudinal Aging Study showed higher education as a positive associate for high NCD risks in men and a reverse U-pattern in women ( i.e., low risk profile for non-educated, higher for middle-educated, then declining risk for higher education) [35]. Gender and occupational status of individuals were mapped out to be a associates of high NCD risk profile in bivariate analysis. Individuals engaged in income-generating activities (nearly 29 out of 50 individuals) and males (17 out of 29 male subjects) had a higher proportion in the high NCD-risk groups. Nevertheless, these associations were not significant in a multivariate model. Nevertheless, these associations were not significant in a multivariate model. Education or other behavioral factors might absorb these effects. Despite the proven genetic basis, family history was not associated with high-risk status (15 of 28 individuals) [53]. It could stem from recall bias, lack of knowledge of family medical history, or sequential contribution of other risk factors that overrode the statistical effect.

The findings of the study call for prioritizing actions for identifying high-risk subjects on the basis of their personal attributes. A change in mindset is required for the recognition of the growing menace of NCDs, even in illiterates.

Limitations and future recommendations

Valuable insights provided by this study are accompanied by certain limitations. The cross-sectional design precludes establishing causal relationships between NCD development and associated risk factors. Recall or social desirability bias may have influenced self-reported data. The present study focused primarily on behavioral and anthropometric indicators owing to the community-based field setting and logistic constraints, which limited the inclusion of biochemical assessments. Additionally, the non-use of objective standardized tools (e.g., Global Physical Activity Questionnaire (GPAQ) or the International Physical Activity Questionnaire (IPAQ)) for assessing physical activity is also to be acknowledged. The findings may also lack generalizability due to geographical and programmatic variations. Future longitudinal studies are recommended to elucidate causal pathways and identify critical windows for effective intervention. Inclusion of biochemical indicators in future studies is also suggested to enhance the analytical depth. Overall, the findings underscore the urgent need for a multisectoral, culturally tailored preventive framework that concurrently addresses the full constellation of risk factors rather than focusing on individual risks in isolation.

## Conclusions

The Northern Indian population in this community-based study was at a vital health transition phase. The high clustering and pervasiveness of behavioral and intermediate NCD risk factors among young subjects is an emerging health challenge. It questions culturally ingrained assumptions regarding healthy rural habits and activity levels. The rich influence of age, education, gender, occupation, and other social determinants on risk status calls for a systematic shift from singular interventions to a holistic approach, including socio-culturally attuned policy advocacy.
